# Lead Contamination of Surgical Gloves by Contact with a Lead Hand

**DOI:** 10.5402/2011/946370

**Published:** 2011-12-07

**Authors:** A. Mehra, D. E. Deakin, A. Khan, T. M. T. Sheehan, P. Nightingale, S. C. Deshmukh

**Affiliations:** Department of Orthopaedics and Clinical Biochemistry, City Hospital, Birmingham B18 7QH, UK

## Abstract

*Background*. “Lead hands” are frequently used to maintain hand and finger position in hand surgery. The malleability and strength of lead make it ideal for this purpose. The aim of this study was to determine the amount of lead transferred to a surgeon's glove during handling of a lead hand. *Method*. Sterile surgical gloves were wiped over the surface of a lead hand. The number of wipes was varied, the gloves were then sent to a trace elements laboratory, and the lead content transferred to each glove was determined. *Results*. The amount of lead transferred to each glove increased with increasing exposure to the lead hand. After twenty wipes, up to 2 mg of lead was transferred to the surgeon's glove. Covering the lead hand with a sterile drape markedly reduced the lead transferred to the surgeon's glove. *Conclusion*. Significant amount of lead is transferred on to the gloves after handling a lead hand. This risks wound contamination and a foreign body reaction. Covering the lead hand with a sterile drape may minimise the risk of surgical wound contamination.

## 1. Introduction

Lead hands are frequently used to maintain the position of a limb during hand surgery [1]. They are easily constructed from lead sheets. Lead's malleability and strength make them ideal for this purpose [2, 3]. Lead oxide residue from the lead hand is frequently transferred to surgical gloves after handling the device (Figure 4). To date, there is no published data describing the amount of lead transferred to the surgeon's gloved hands after contact with a lead hand. We hypothesised that handling a lead hand results in lead contamination of the surgeon's gloves. There is, hence, a potential risk that some of these lead particles may contaminate the surgical wound causing an inflammatory (i.e., foreign body) reaction. The aim of this study was to determine the lead concentration on surgical gloves after handling a lead hand.

## 2. Methods

A sterile surgical glove (Biogel, Mölnlycke Healthcare, Gothenburg, Sweden) was opened and worn over the investigators right hand (AM). The investigators gloved hand was then wiped over the surface of a lead hand (Integra Miltex) in a smooth and reproducible manner. The number of wipes was increased sequentially and each glove was removed and sealed in a plastic container separately. The gloves were transferred to the local trace elements laboratory and placed in a 120 mL polypropylene, screw-cap container (Sarstedt, UK), and 50 mL of 0.15% HNO3 were added. The container was capped securely and then rolled for 2 hours to aid dissolution of the lead. The acid was then analysed for lead (208 isotope) by inductively coupled plasma mass spectrometry using an Agilent 7500c (Agilent, UK) and with rhodium as an internal standard. Calibration standards were prepared in 0.15% HNO3, and this reagent was also used to dilute the glove washings when necessary. Lead content on each glove was determined. The gloves which were not wiped across the lead hand acted as a control. 

In a second experiment, a glove was wiped over a different lead hand ten times. A second glove was then wiped over the same area a further ten times, and this procedure was repeated for a total of ten gloves. This aimed to determine the effect of repeated handling of the same area of the lead hand on lead content over the surface of the surgical gloves.

In a further experiment, the lead hand was sandwiched between two sterile OPSITE (Smith and Nephew) drapes, and experiment one was repeated (Figure 5). This aimed to determine the amount of lead transferred to the glove after minimising its contact with the lead hand with an OPSITE drape.

## 3. Results

A glove not wiped across the lead hand acted as a control, and 2 *μ*g of lead was recovered. The amount of lead recovered from a glove wiped five times across the lead hand was 875 *μ*g. The amount of lead increased proportionally with the increase in the number of wipes (Table 1, Figure 2).

**Figure 1:**
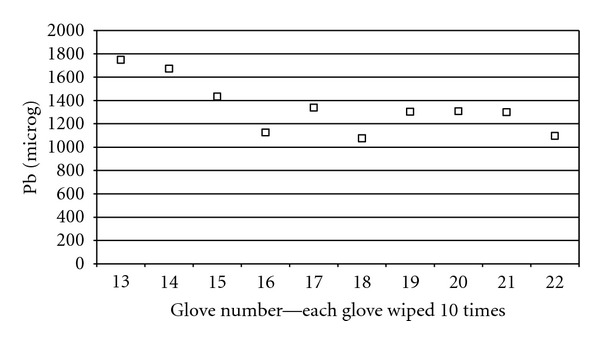
Lead recovered from each glove.

**Figure 2:**
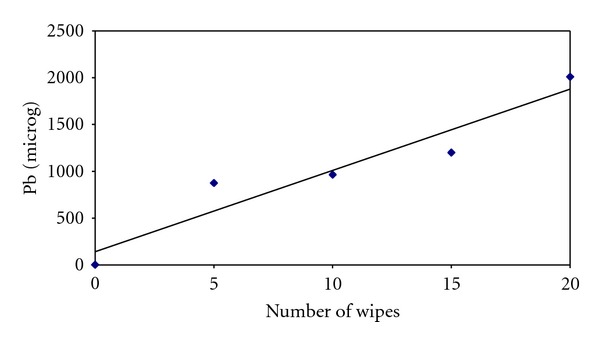
Regression line for Table 1 data.

When gloves were wiped repeatedly over the same area of a different lead hand the amount of lead recovered was maximum after the first ten wipes (1749 *μ*g) (Table 2, Figure 1). This amount reduced slightly with subsequent wipes but then remained stable between 1076 *μ*g and 1339 *μ*g each time a glove was wiped over the same area ten times. 

**Figure 3:**
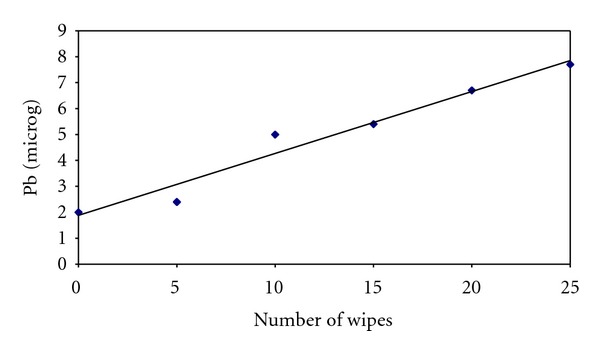
Regression line for Table 3 data.

**Figure 4:**
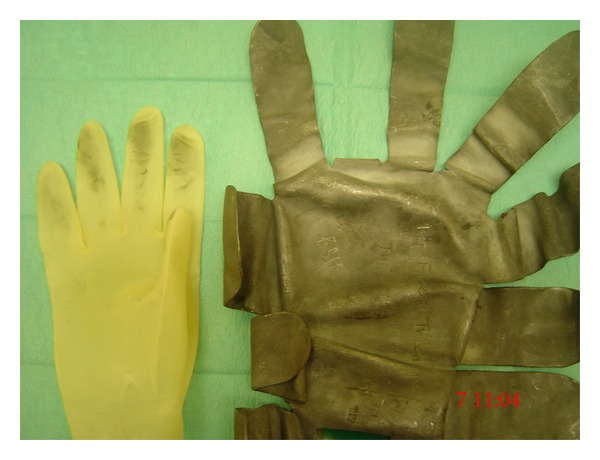


**Figure 5:**
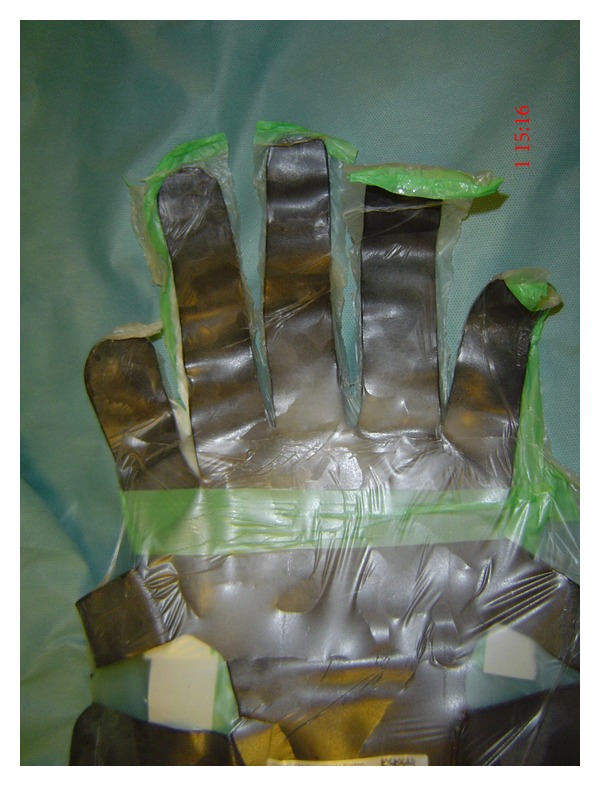


With the use of OPSITE (Smith and Nephew) over the lead hand the amount of lead contamination rose minimally from 2 *μ*g to 7.7 *μ*g after twenty-five wipes (Table 3, Figure 3). But, the increase was significantly lower than the increase seen without the impervious cover. 

Straight line graphs were plotted for experiment 1 and 3 and the slopes estimated. For experiment 1 (Table 1), the slope was 86.8 micrograms per wipe (95% confidence interval is 36.4–137.2), and for experiment 3 (Table 3), the slope is 0.24 micrograms per wipe (95% confidence interval is 0.17–0.31).

## 4. Discussion

The malleability and strength of lead make it an ideal material for use in a lead hand [4]. However, lead is a highly toxic element, and ingestion or administration to humans has been associated with a number of serious side effects [5]. Normal blood lead concentrations are below 0.48 *μ*mol/L. Experiments looking at percutaneous absorption of inorganic lead compounds have shown no increase in total lead in blood or urine [6, 7]. While no evidence exists that use of a lead hand is associated with lead poisoning in patients, our study shows that up to 2 mg of lead can be transferred to a surgeons glove by direct contact with the lead hand. This amount may be double if both the surgeon's gloves are used to handle the lead hand. Handling the surgical wound may cause some of this lead to contaminate the patient's tissues. The effect of this potential lead contamination both locally and systemically is unknown but may be the cause of florid inflammatory (i.e., foreign body) reaction seen in some patients.

Our study demonstrates that the amount of lead contamination of the surgeon's gloves is directly related to the amount of contact with the lead hand. The lead contamination of surgeons gloves can be minimised by covering the lead hand with an impervious plastic drape. Surgeons using lead hands should be aware of this lead contamination and also of the fact that this risk can be minimised by covering the lead hand with an impervious drape. Newer malleable plastic hands may also solve this problem.

We did not measure the blood lead levels in our patients, as inorganic lead absorption through the skin has been found to be essentially zero [7]. Another interesting observation made during the study was that the lead content in the first experiment after 10 wipes was much lower than the lead levels found in the second experiment after 10 wipes. This may be related to the age of the lead hand with the older lead hand shedding more lead even though this is difficult to prove.

## 5. Conclusion

The study has confirmed our hypothesis that handling a lead hand during surgery results in deposition of lead on the surgeon's gloves. This may be responsible for the wound inflammation seen occasionally in patients. This risk can be minimised by covering the lead hand with an impervious drape. Further studies are required to provide more robust evidence.

## Figures and Tables

**Table 1:** Lead recovered from each glove with increasing number of wipes across a lead hand.

Glove number	Number of wipes across a lead hand	Pb (*μ*g)
Glove 1 (control)	0	2
Glove 2	5	875
Glove 3	10	965
Glove 4	15	1200
Glove 5	20	2010

**Table 2:** Lead recovered from each glove after repeated exposure to the same area of the lead hand.

Glove number	Number of wipes	Pb (*μ*g)
Glove 12	0	2
Glove 13	10	1749
Glove 14	10	1673
Glove 15	10	1434
Glove 16	10	1126
Glove 17	10	1339
Glove 18	10	1076
Glove 19	10	1304
Glove 20	10	1308
Glove 21	10	1300
Glove 22	10	1096

**Table 3:** Lead recovered from each glove with increasing number of wipes across a lead hand covered by an impervious sterile plastic drape.

Glove number	Number of wipes across a lead hand protected by a plastic sterile drape	Pb (*μ*g)
Glove 6 (control)	0	2
Glove 7	5	2.4
Glove 8	10	5
Glove 9	15	5.4
Glove 10	20	6.7
Glove 11	25	7.7
